# A national survey of Canadian ophthalmologists to determine awareness of published guidelines for the management of uveitis

**DOI:** 10.1186/s12348-016-0102-3

**Published:** 2016-10-18

**Authors:** Crystal S. Y. Cheung, Nima Noordeh, Chloe C. Gottlieb

**Affiliations:** 1 Faculty of Medicine, University of Ottawa, Ottawa, Ontario Canada; 2 University of Ottawa Eye Institute, Ottawa, Ontario Canada; 3 Ottawa Hospital Research Institute, Ottawa, Ontario Canada; 4 Department of Ophthalmology, Kensington Eye Institute, 340 College Street, Suite 600, Toronto, ON M5T 3A9 Canada

**Keywords:** Uveitis, Guidelines, Management

## Abstract

**Background:**

The objectives of this study are to assess Canadian ophthalmologists’ awareness of established uveitis treatment guidelines and clinical management of uveitis and to assess the frequency of government applications for immunomodulatory therapy (IMT) and identify primary prescribers.

A 25-item questionnaire was sent to 759 practicing Canadian ophthalmologists. Six questions assessed demographics including the year of residency completion, training by uveitis specialists during residency, and fellowship training. Five questions assessed application of guidelines to clinical scenarios, and 12 questions assessed referral patterns and success of obtaining coverage for IMT.

**Results:**

Of 144 respondents, 12 (8.3 %) were uveitis specialists; 45.1 % of respondents had uveitis training during residency by a uveitis specialist. Sixty-one percent reported awareness of management guidelines. Recent graduates (2001–2012) referred patients to uveitis specialists (55.3 %) less frequently than earlier graduates. Recent graduates also managed uveitis patients more frequently with corticosteroid injections (15.6 %) than those who graduated before 1980 (9.75 %). The majority (93.6 %) of respondents submitted less than six IMT funding applications for provincial drug coverage yearly, and 5.5 % reported prescribing IMT themselves, rather than referring to other specialists.

**Conclusions:**

Although greater than half of respondents reported awareness of uveitis treatment guidelines, Canadian ophthalmologists’ awareness of uveitis treatment guidelines and application of the guidelines to patient care could be improved. Few applications are made for IMT, and the majority of applications are sent by non-ophthalmologists. This suggests the need for further education of ophthalmologist about uveitis treatment guidelines and for more ophthalmologists trained to manage uveitis with IMT.

**Electronic supplementary material:**

The online version of this article (doi:10.1186/s12348-016-0102-3) contains supplementary material, which is available to authorized users.

## Background

Uveitis, a group of ocular inflammatory conditions, accounts for 10 to 15 % of blindness in developed countries [1, 2]. As per standard nomenclature, chronic uveitis is defined as persistent uveitis characterized by prompt relapse (in less than 3 months) after discontinuation of therapy [3]. Persistent uveitis is defined as inflammation greater than 3 months in duration [3]. Recurrent uveitis is repeated episodes of uveitis separated by periods of inactivity without treatment, in which these periods of inactivity without treatment are at least 3 months in duration [3]. Sight-threatening disease arises from chronic, persistent, or recurrent inflammation. Left untreated or undertreated, chronic, persistent, or recurrent inflammation may lead to complications, such as cataract, glaucoma, macular edema, retinal or choroidal inflammation and atrophy, intraocular hemorrhage, or optic nerve atrophy. The therapeutic goal is to prevent vision loss from these complications by controlling or eliminating chronic, persistent, or recurrent inflammation. Systemic corticosteroid and locally administered corticosteroid injections treat ocular inflammation during acute episodes of uveitis, but the side effects of multiple pulse treatments or prolonged systemic corticosteroid administration limit their convenience as monotherapy for chronic and persistent uveitis, except in selected cases where dexamethasone or fluocinolone implants have been shown to be successful as monotherapy [4]. Persistent or chronic cases of uveitis, where corticosteroid therapy has failed to adequately treat disease, require long-term immunomodulatory therapy (IMT) to treat active inflammation and reduce the frequency of recurrences [5]. The uveitis management guidelines developed by an international panel of experts recommends the initiation of corticosteroid-sparing IMT if intraocular inflammation cannot be controlled with <10 mg/day of prednisone within 3 months [5]. However, in the recent survey of uveitis treatment patterns in the USA, Nguyen et al. showed that 75 % of the physician cohort surveyed was not aware of the aforementioned management guidelines [6]. The survey also indicated that the average corticosteroid dose was 44 mg/day and this dose was maintained for an average duration of 21 months, which is much higher than the recommended tapered doses of corticosteroids to <10 mg/day within 3 months [5]. The high doses of corticosteroid contributed to the significant adverse effects observed in the cohort of uveitis patients in the survey. Therefore, more widespread awareness of the uveitis treatment guidelines, leading to less reliance on corticosteroids, and appropriate timing of corticosteroid-sparing IMT would likely improve patient outcomes.

The purpose of this study is to assess, among Canadian ophthalmologists, the current awareness of published guidelines for the treatment of uveitis and to evaluate whether practice patterns are congruent with the published uveitis treatment guidelines. This study also assessed the frequency of applications to the government and private insurance providers for immunomodulatory drugs or biologics.

## Methods

### Survey population and development

The study was approved by the Ottawa Health Science Network Research Ethics Board. A 25-item questionnaire was developed by the authors and distributed electronically to 759 practicing Canadian ophthalmologists via the Canadian Ophthalmological Society (COS). Residents and fellows were not included in this study. Informed consent was implied with the completion and electronic submission of the survey. No remuneration was given to respondents. Data was collected over a 4-month period from August 2012 to November 2012.

The following data were collected: the year of residency completion, presence of a uveitis specialist at the respondent’s center of residency training, fellowship training in uveitis, and other sub-specialities including pediatric ophthalmology, retina, anterior segment and others, and percentage of patients with uveitis in the respondent’s practice. Practice pattern data was also collected. This included awareness and utilization of uveitis treatment guidelines, referral patterns and associated barriers to referrals, co-management of uveitis with uveitis specialists or non-ophthalmologists, number of IMT applications for provincial and private insurance drug coverage, and success of obtaining IMT from these providers. Understanding and application of the aforementioned uveitis treatment guidelines was assessed by survey questions comprising definitions and clinical scenarios (Additional file 1).

### Data analysis

Demographic and other data were tabulated. The proportion of total correct responses to the clinical scenario questions was defined as the proportion of cumulative correct responses to each question. Non-responses were not included in the proportions. Descriptive analyses were conducted where appropriate, and confidence interval (CI) for proportions was calculated using the exact method.

Statistical analysis was performed using SAS software (SAS Institute Inc., Cary, NC). Analyses were considered to be significant if *p* < 0.05; reported *p* values were not adjusted for multiple testings and should be considered nominal. Use of Fisher’s exact test and chi-squared test was based on sample sizes. Fisher’s exact test was used to determine whether awareness of uveitis management guidelines differed according to the following training circumstances: (1) sub-specialty training, (2) fellowship training in uveitis, and (3) presence of a uveitis specialist at the respondents’ center of residency training. Chi-squared test was used in the comparison of the proportion of total correct responses to clinical scenario questions to the following variables: (1) sub-specialty training, (2) fellowship training in uveitis, and (3) presence of a uveitis specialist at the respondents’ center of residency training.

The year of residency completion was categorized into three groups: (1) 1960 to 1980, (2) 1981 to 2000, and (3) 2001 to 2012. Multivariate analysis was used to determine whether the year of residency completion was associated with the proportion of total correct responses to clinical scenario questions. Descriptive analyses were used to compare the year of residency completion with the frequency of selecting “corticosteroid injections” as response to clinical scenario questions and frequency of referral to uveitis specialists.

## Results

The response rate of the survey was 19.23 % (146/759), and 87.7 % (128/146) of respondents completed the survey in its entirety. Demographic and referral pattern data are detailed in Table 1. Among the respondents, 8.3 % (12/146; CI = 0.04–0.14) were uveitis specialists. A fellowship-trained uveitis specialist was present at 45.8 % (66/148; CI = 0.36–0.53) of the respondents’ center of residency training. Overall, 93.8 % (137/146; CI = 0.89–0.97) of the physicians’ practices consisted of less than 30 % of uveitis patients. The majority of respondents (73.3 %, 107/146; CI = 0.65–0.80) indicated they have referred patients to uveitis specialists.

**Table 1:** Demographics and referral pattern data of respondents

Question	*n* (%)	CI
Type of clinical practice		
Community practice	91 (63.2)	0.55–0.71
Part-time academic	21 (14.6)	0.09–0.21
Full-time academic	32 (22.2)	0.16–0.30
No response	2	
Year of ophthalmology residency completion		
1960–1970	7 (4.8)	0.02–0.09
1971–1980	23 (15.8)	0.10–0.23
1981–1990	43 (29.5)	0.22–0.37
1991–2000	34 (23.3)	0.17–0.31
2001–2012	39 (26.7)	0.18–0.35
No response	0	
Sub-specialty or fellowship training		
No fellowship training	67 (45.9)	0.35–0.51
Retina	24 (16.4)	0.10–0.22
Pediatrics	11 (7.5)	0.04–0.12
Uveitis	12 (8.2)	0.04–0.13
Cornea/anterior segment	15 (10.3)	0.05–0.15
Other	27 (18.5)	0.12–0.24
No response	0	
Fellowship-trained uveitis specialist present at the respondents’ center of residency training		
Yes	66 (45.8)	0.37–0.54
No	78 (54.2)	0.46–0.62
No response	2 (1.37)	
Percentage of patients with uveitis in respondents’ clinical practice		
>60 %	2 (1.4)	0.002–0.05
30–60 %	7 (4.8)	0.02–0.09
<30 %	137 (93.8)	0.89–0.97
No response	0	
Referral to uveitis specialists		
Yes	107 (73.3)	0.65–0.80
No	27 (18.5)	0.13–0.26
Respondent is a uveitis specialist	12 (8.2)	0.04–0.14
No response	0	
Barriers of referral encountered when referring patients to uveitis specialist		
Geography (distance)	38 (32.2)	0.19–0.34
Wait time	46 (39.0)	0.24–0.39
None	40 (33.9)	0.20–0.35
Not application. Care for patients with uveitis	15 (12.7)	0.06–0.16
Other	8 (6.78)	0.02–0.10
No response	28	

*n* number of respondents

Self-reported awareness of the uveitis management guidelines was 61.0 % (89/146; CI = 0.53–0.69), and 51.7 % (74/143; 3 no responses; CI = 0.43–0.60) of respondents indicated the utilization of these guidelines. The proportion of total correct responses to the clinical scenario questions was not statistically different (*p* = 0.547) between physicians who reported awareness of the aforementioned guidelines and those who did not. Awareness of guidelines did not differ significantly between respondents who were trained in uveitis (*p* = 0.127), respondents with sub-speciality training (*p* = 0.179), and presence of a fellowship-trained uveitis specialist at the respondent’s center of residency training (*p* = 0.232) (Table 2).

**Table 2:** Awareness of uveitis management guidelines in association with different training circumstances

Awareness of treatment guidelines	Aware, *n* (%)	Not aware, *n* (%)	*p* value	CI
Stratified by the presence of a fellowship-trained uveitis specialist at the center of residency training	89 (50.0 %)	57 (39.3)	0.232	−0.057 to 0.26
Stratified by fellowship training in uveitis	10 (83.30 %)	2 (16.70 %)	0.127	NA, *n* < 5
Stratified by fellowship training in a related sub-specialty	50 (64.96 %)	27 (35.06 %)	0.179	−0.05 to 0.28

*n* number of respondents, *CI* confidence interval, *NA* not applicable

Adherence to the uveitis management guideline recommendations [1] was evaluated using clinical scenario questions. The association between adherence to guidelines and different training circumstances was assessed. A statistically significant difference in the proportion of total correct responses to clinical scenario questions was also found between those with fellowship training in sub-specialities and those with no fellowship training (*p* = 0.0145). However, the proportion of total correct responses to the clinical scenario questions was not statistically significant across the following groups of comparisons: whether the respondent had fellowship training in uveitis (*p* = 0.0509) and whether a uveitis specialist was present at the respondent’s center of residency (*p* = 0.698) (Table 3).

**Table 3:** Total response rate to uveitis management questions in association with awareness of treatment guidelines, different training circumstances, and the year of residency completion

Stratification	Number of responses, *N*	Number of correct responses, *n* (%)	95 % Confidence interval	*p* value
Awareness of uveitis treatment guidelines				
Aware	406	242 (59.61)	0.55–0.64	0.5471
Not aware	243	129 (53.09)	0.47–0.59	
Fellowship training				
Fellowship training	348	189 (54.31)	0.49–0.60	0.0145
No fellowship training	301	192 (63.79)	0.58–0.69	
Uveitis fellowship training				
Uveitis-trained	56	26 (46.43)	0.33–0.60	0.0509
No uveitis training	593	355 (59.87)	0.56–0.64	
Uveitis specialist present at the center of the respondents’ residency training				
Uveitis specialist present	288	166 (57.64)	0.52–0.63	0.6981
No uveitis specialist present	355	210 (59.15)	0.54–0.64	
Year of residency completion				
1960–1980	131	84 (64.12)	0.55–0.72	1981–2000 vs 1960–1980: 0.3596
1981–2000	352	214 (60.80)	0.55–0.66	2001–2012 vs 1981–2000: 0.0208
2001–2012	166	83 (50)	0.42–0.58	2001–2012 vs 1960–1980: 0.0067

*vs* versus

**Fig. 1:**
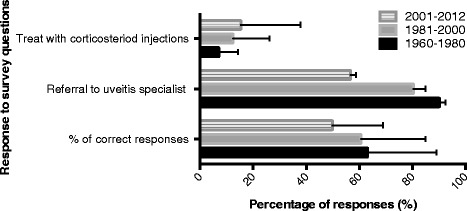
The year of residency completion in association with the (a) proportion of total correct responses to clinical scenario questions (*p* = 0.0208 for 2001–2012 vs 1981–2000; *p* = 0.0067 for 2001–2012 vs 1960–1980), (b) frequency of referral to uveitis specialists, and (c) frequency of managing patients with corticosteroid injections in clinical scenario questions

The year of residency completion was categorized into three groups: 1960 to 1980, 1981 to 2000, and 2001 to 2012. The proportion of total correct responses to clinical scenario questions in association with the year of residency completion is detailed in Table 3. When comparing the proportion of total correct responses in clinical scenario questions to the year of residency completion, there was a statistically significant difference between respondents who completed residency during 2000 to 2012 and those during 1960 to 1981 (*p* = 0.0208) and 1981 to 2000 (*p* = 0.0067). The selection of corticosteroid injections (intravitreal or periocular) in the clinical scenario questions, which was an incorrect response, was 7.1 % among graduates from 1960 to 1980, 12.4 % in graduates from 1981 to 2000, and 15.6 % in graduates from 2001 to 2012. Referral to a uveitis specialist was 90.0 % (27/30; CI = 0.73–0.98) among those who completed residency during 1960 to 1980, 76.6 % (59/77; CI = 0.63–0.83) for graduates in 1981 to 2000, and 53.8 % (21/39; CI = 0.37–0.70) in 2001 to 2012 (Fig. 1).

The frequency of IMT applications and success of obtaining IMT are shown in Table 4. When IMTs are required, 5.5 % of physicians (8/145; 1 no response; CI = 0.02–0.11) would prescribe the IMT themselves, while 54.5 % (79/145; CI = 0.46–0.63) would seek prescription of the IMT from a rheumatologist, dermatologist, or general internist, and 40.0 % (58/145; CI = 0.32–0.48) would refer to an ophthalmologist to coordinate the acquisition of IMT. Overall, 93.6 % (117/125; 21 no responses; CI = 0.88–0.97) of physicians make less than six IMT applications per year, and 89.0 % (105/118; 28 no responses) of respondents make less than six co-IMT applications with rheumatologists or other physicians. Less than 10 % of IMT applications was successfully obtained through private insurance and provincial drug coverage among 64.0 % of physicians (71/111; 35 no responses; CI = 0.54–0.73) and 56.5 % of physicians (61/108; 38 no responses; CI = 0.47–0.66), respectively. The frequency of IMT applications was 25.0 % (17/68; 78 no responses; CI = 0.15–0.37) for mycophenolate mofetil, 38.2 % (26/68; CI = 0.27–0.51) for cyclosporine, 32.4 % (22/68; CI = 0.22–0.45) for anti-tumor necrosis factor (TNF), and 36.8 % (25/68; CI = 0.25–0.49) for others.

**Table 4:** Immunomodulatory therapy applications to public and private insurance providers

Question	*n* (%)	95 % CI
How do you obtain immunomodulatory drugs or biologics for your patients?		
Request from a rheumatologist, dermatologist, gastroenterologist, or internist	79 (54.5)	0.46–0.62
Refer to an ophthalmologist who will prescribe or coordinate further care for patients with uveitis	58 (40.0)	0.32–0.48
Prescribe yourself	8 (5.5)	0.02–0.11
No response	1	
Refer patients to a uveitis specialist		
Yes	107 (73.3)	0.65–0.80
No	27 (18.5)	0.13–0.26
I am a uveitis specialist	12 (8.2)	0.04–0.14
No response	0	
Number of new applications made to the government per year for IMT coverage		
0–5	117 (93.6)	0.88–0.97
6–10	5 (4.0)	0.01–0.09
11–20	2 (1.6)	0.002–0.06
21–30	1 (0.8)	0.0002–0.04
>30	0	
No response	21	
Number of co-applications for IMT made with rheumatologists or other physicians		
0–5	105 (89.0)	0.69–0.84
6–10	10 (8.5)	0.03–0.13
11–20	2 (1.7)	0.002–0.05
21–30	0	0.00–0.03
>30	1 (0.8)	0.002–0.04
No response	28	
Percentage of respondent’s patients with their IMT successfully covered through government or provincial health plan		
<10 %	71 (64.0)	0.54–0.73
10–50 %	32 (28.8)	0.21–0.38
51–90 %	5 (4.5)	0.02–0.10
>91 %	3 (2.7)	0.006–0.08
No response	35	
Percentage of respondent’s patients with their IMT successfully covered through private insurance		
<10 %	61 (56.5)	0.46–0.66
10–50 %	29 (26.9)	0.19–0.36
51–90 %	10 (9.3)	0.04–0.16
>91 %	8 (7.4)	0.03–0.14
No response	38	
Class of IMT applied through the Exceptional Access Program		
Mycophenolate mofetil	17 (25.0)	0.11–0.28
Anti-TNF	22 (32.4)	0.16–0.35
Cyclosporine	26 (38.2)	0.20–0.39
Others	25 (36.8)	0.19–0.38
No response		

*IMT* immunomodulatory therapy, *TNF* tumor necrosis factor

## Discussion

Despite the well-recognized adverse effects of long-term corticosteroid usage, it has been the mainstay of uveitis management since its development [5, 7]. In addition to concerns about their adverse effects, high-dose systemic or locally injected corticosteroids, particularly serial periocular or intraocular corticosteroid injections, are not an optimal management strategy for chronic or frequently recurrent uveitis. At the time of writing, no Health Canada-approved immunomodulatory drugs are indicated for uveitis and all such drugs are used off-label. A sustained-release dexamethasone intravitreal implant (Ozurdex; Allergan, Inc., Irvine, CA, USA) and Retisert (Bausch&Lomb, Rochester, NY, USA) are currently the only Health Canada-approved treatments for uveitis. However, these implants are only indicated for posterior uveitis and not anterior or intermediate uveitis [5]. Although studies have demonstrated the effectiveness of Ozurdex for non-infectious posterior uveitis, the protracted disease course of some patients with chronic uveitis, measured in years and decades, is lengthy compared to the 6-month duration of a steroid implant [8]. When treatment with systemic corticosteroid and/or multiple local injections or implants have failed to reduce intraocular inflammation to an acceptable level, then corticosteroid-sparing IMT is warranted. An international expert panel has published guidelines recommending the appropriate initiation of corticosteroid-sparing IMT. [5]

To the best our knowledge, this is the first study examining the awareness of and adherence to uveitis management guidelines among Canadian ophthalmologists. This study described the awareness and utilization of the aforementioned guidelines, practice pattern variations across different years of residency completion, and the frequency of prescribing IMT and co-management of uveitis patients.

### Awareness of and adherence to uveitis management guidelines

In this survey, self-reported awareness of the uveitis management guidelines was 61.0 % (CI = 0.53–0.69) and 51.7 % (CI = 0.43–0.60) of respondents indicated utilization of these guidelines. The level of awareness of the aforementioned guidelines found in this study is higher than those found by Nguyen et al., where 25 % of the US physician cohort surveyed was aware of the aforementioned management guidelines [4, 5]. In addition, the results of this survey showed that self-reported awareness of guidelines was not associated with significantly higher proportion of total correct responses to the clinical scenario questions (*p* = 0.547), suggesting that ophthalmologists who reported awareness of the uveitis management guidelines did not use the guidelines in their clinical practice.

Factors which influence respondents’ awareness of treatment guidelines were assessed. Awareness and unawareness of guidelines did not differ significantly for respondents who were trained in uveitis (*p* = 0.127), respondents with any sub-speciality training (*p* = 0.179), and presence of fellowship-trained uveitis speciality at the respondent’s center of residency training (*p* = 0.232) (Table 2). These cumulative results suggest that awareness of published recommendations was not affected by sub-specialty training (including uveitis training) or residency training by a uveitis specialist. Despite the large difference observed between self-reported awareness (83.30 %; CI not applicable) and unawareness (16.70 %; CI not applicable) of management guidelines among the uveitis specialists, no statistically significant difference was found. This may be attributable to inadequate power of the analysis due to the small number of uveitis specialists (12) who responded to this survey.

The associations between adherence to guidelines and different training circumstances were assessed. Adherence to guideline recommendations was determined by the proportion of total correct responses to clinical scenario questions. The proportion of total correct responses was significantly lower for respondents who had previous sub-specialty fellowship training than those who did not (*p* = 0.0145), suggesting that respondents with comprehensive ophthalmology practices were more likely to correctly identify cases where referral for IMT is necessary. Initially, this finding appears to contradict our hypothesis that additional fellowship training may be associated with higher likelihood of correctly identifying responses in clinical scenario questions. This apparent inconsistency may be due to the complexity of the cases presented in the clinical scenario questions, which focused on the management of chronic or recurrent uveitis. Multiple studies have shown that community-based comprehensive ophthalmologists manage a greater proportion of anterior uveitis and responsive uveitis cases than the ophthalmologists in university referral centers [9–11]. Uveitis cases which are refractory to treatment or chronic are more frequently managed by university-based ophthalmologists, most frequently uveitis specialists [9–11]. Since community-based ophthalmologists are more likely to refer treatment-refractory patients to specialists, this may explain the greater proportion of total correct responses observed in this study.

In contrast, the proportion of total correct responses was not significantly different between uveitis specialists and those without uveitis fellowship training (*p* = 0.0509). This analysis may not be accurate due to the limited number of uveitis specialists included in this study, and as such, the results are inconclusive. The proportion of total correct responses was also not significantly higher between respondents who had a uveitis specialist present at the academic center where they completed residency training and those who did not (*p* = 0.6981). As such, ophthalmology residents may have limited interactions with these specialists.

### Uveitis management patterns among the most recent graduates

The year of residency completion was associated with a lower proportion of total correct responses. In particular, the most recent graduates (i.e., completed residency between 2001 and 2012) were less likely to correctly answer the clinical scenario questions compared to respondents who completed residency from 1960 to 1980 (*p* = 0.0067) and from 1981 to 2000 (*p* = 0.0208). This suggests the most recent graduates were less likely to correctly identify uveitis cases where IMT induction is necessary based on the management guidelines [5]. In the 2008 Canadian survey evaluating residents and recent graduates’ perception of competencies in different areas of clinical training, the sub-specialties where most respondents reported insufficient exposure were low-vision rehabilitation (77.5 %), refraction and glasses prescription (65 %), and neuro-ophthalmology (45 %); only approximately 15 % of respondents indicated that clinical exposure to uveitis was inadequate in achieving competency [12]. The study by Zhou et al. described “adequate clinical exposure” broadly and did not specify exposures to investigations, diagnosis, or management strategies [12]. In contrast, the clinical scenario question in this survey focused on uveitis management, particularly the appropriate initiation of corticosteroid-sparing IMT and appropriate timing of referral to a uveitis specialist for chronic uveitis management. Nonetheless, the discrepancy between recent graduates’ perception of competence in uveitis and their lower proportion of correct responses to clinical scenario questions suggests the importance of raising awareness of uveitis management guidelines in Canadian residency programs.

Self-reported referral rate to a uveitis specialist was the lowest among the most recent graduates (53.8 %; CI = 0.37–0.70) compared to those who completed residency during 1960 and 1980 (90.0 %; CI = 0.73–0.98) and during 1981 to 2000 (76.6 %; CI = 0.66–0.85). The lower rate of referrals among newer graduates found in this survey parallels the results of the 2004 Canadian National Uveitis Survey [13]. In addition to differences in the proportion of referrals, recent graduates were also more likely to manage IMT-requiring uveitis with off-label corticosteroid injections. This observation may be attributable to the rapid increase of intravitreal injections performed for posterior segment diseases in the past decade [14, 15]. In this case, the increased likelihood to treat uveitis with corticosteroid injections among the most recent graduates may contribute to the lower proportion of referrals observed.

### Applications for immunomodulatory therapy coverage to provincial drug coverage and private providers

Immunomodulatory agents are indicated for corticosteroid-sparing in patients with recurrent or chronic ocular inflammation where the dose of systemic corticosteroid is ≥10 mg/day or ≥0.1 mg/kg/day and duration of corticosteroid therapy is ≥3 months [5, 16]. The adverse effects of corticosteroid therapy have been shown to be more frequent than IMT [17, 18]. However, many ophthalmologists do not have extensive training in IMT administration, and monitoring and collaboration with uveitis specialists, rheumatologists, or internists is necessary [4]. Our results showed that only 5.5 % (CI = 0.02–0.11) of physicians would prescribe IMT themselves and 93.6 % (CI = 0.88–0.97) make less than six IMT applications for provincial and private provider drug coverage per year. Thus, IMT initiation by ophthalmologists is infrequent. When IMT is required, 54.5 % (CI = 0.46–0.63) of respondents would coordinate IMT acquisition with specialists such as rheumatologists, dermatologists, or general internists. However, 89.0 % (CI = 0.82–0.94) of respondents make less than six co-applications for IMT with rheumatologists or other physicians. Cumulatively, these results suggest that ophthalmologists make few IMT applications themselves and co-application with other physicians and majority of IMT applications are sent by non-ophthalmologists.

Over 73 % (CI = 0.65–0.80) of respondents stated that they refer patients to uveitis specialists. When IMT initiation is required, 40.0 % (CI = 0.32–0.48) of physicians would refer patients to uveitis specialists. Although majority of respondents refer patients to uveitis specialists, they also frequently identified geographic distance (32.2 %; CI = 0.24–0.41)) and wait time (39.0 %; CI = 0.30–0.48) as barriers. These barriers to referral may delay the initiation of IMT and result in further vision loss secondary to persistent ocular inflammation. Given the limitations of timely access to uveitis specialists, coordinating the acquisition of IMT with other specialists such as rheumatologists and internists is essential. Yet, the low number of co-IMT applications (fewer than six) made by ophthalmologists with other physicians is concerning.

The uveitis management guidelines outline the circumstances in which IMT induction is necessary [5]. However, there is limited information available on which IMT is considered first-line corticosteroid-sparing agents. Preferences for different IMT classes were assessed in this study. The results showed that the frequency of IMT applications was 25.0 % (CI = 0.155–0.37) for mycophenolate mofetil, 32.4 % (CI = 0.22–0.45) for anti-TNF, 38.2 % (CI = 0.27–0.51) for cyclosporine, and 36.8 % (CI = 0.25–0.49) for others. This shows that the proportion of IMT drugs prescribed by ophthalmologists is similar across different types of IMT. In contrast to our findings, a survey of US uveitis specialists showed that methotrexate was preferred for initial management of uveitis while mycophenolate mofetil was preferred for intermediate and posterior uveitis; the primary reason for not prescribing cyclosporine was safety and tolerability. Another practice pattern survey of US uveitis specialists, Canadian specialists use cyclosporine more frequently, while mycophenolate mofetil was used less often [19].

### Limitations

Several limitations of this study should be noted. First, the survey was set up such that mandatory responses to questions were not required and, therefore, incomplete data was recorded in the form of non-responses, resulting in incomplete data analysis and interpretation. A second limitation of this study is sampling bias, where physicians who responded to the study may feel more proficient in the management of uveitis leading to an overestimation of the proportion of self-reported awareness of guidelines and proportion of total correct responses. Therefore, the management patterns of uveitis among Canadian ophthalmologists may not be accurately represented through this survey. Third, the low number of uveitis specialists included in this study limits the statistical analyses. Fourth, there were many non-responses to questions regarding IMT applications to provincial and private drug coverage and this might have been due to respondents not making any IMT applications and the survey questions did not assess this directly. Lastly, it has been reported that indicators for treatment vary among uveitis specialists despite guidelines, and this variation is even greater among non-uveitis specialists. As such, this may overestimate the observed variability in responses to the survey [20].

## Conclusions

In conclusion, the awareness and utilization of the uveitis management guidelines among Canadian ophthalmologists can be improved. Furthermore, recent graduates were less likely to identify cases of uveitis where treatment with IMT is indicated. They were also more likely to manage uveitis with corticosteroid injections and less likely to refer patients to uveitis specialists. When use of IMT is necessary, the majority of ophthalmologists indicated that they would refer patients to uveitis specialists or other internists such as rheumatologists and dermatologists. Few applications for IMT were made by ophthalmologists, and majority of these applications were sent by non-ophthalmologists. Cumulatively, these results suggest the need for further education of Canadian ophthalmologists regarding uveitis treatment guidelines and the need to increase the number of uveitis specialists in Canada.

## Additional file

Additional file 1: Clinical scenarios in the uveitis survey. (doc 13 kb)
