# Supplementary material for: A national survey of Canadian ophthalmologists to determine awareness of published guidelines for the management of uveitis
Source: J Ophthalmic Inflamm Infect. 2016 Oct 18;6:38. doi: 10.1186/s12348-016-0102-3 (PMC5069221; doi:10.1186/s12348-016-0102-3)
Supplement: Additional file 1: — Clinical scenarios in the uveitis survey. (doc 13 kb) [file 12348_2016_102_MOESM1_ESM.docx]

**Additional file 1: Clinical scenarios in uveitis survey**

Which of the following would require initiation of immunomodulatory therapy at the time of diagnosis? (Multiple selections permitted)

1. Serpiginous choroidopathy
2. Necrotizing scleritis
3. Mucous membrane pemphigoid
4. Behcet’s disease

A patient diagnosed 3 years ago with birdshot retinochoroiditis has chronic vitreous cells (1+) and haze (1+) OU. The visual acuity is 20/20 OU and the intraocular pressures are normal. Appropriate therapy would be:

1. Observation
2. Topical corticosteriod
3. As-needed intravitreal injections of steroid
4. Immunomodulatory therapy

How would you treat a patient with HLA-B27-positive recurrent anterior uveitis who had 4 flare-ups of uveitis in the past year and now has decreased vision secondary to macular edema?

1. Topical steroid for uveitis and topical NSAID for macula edema
2. Intravitreal or peri ocular injections of steroid
3. Intravitreal steroid implant
4. Refer to specialist for consideration of immunodulatory therapy or other treatments

A patient with pars planitis has chronic vitreous cells (1+) and haze (1+) bilaterally. He is on prednisone (20 mg/day) for the last 3 months with no improvement. You would:

1. Prescribe topical corticosteriod q1h bilaterally
2. Perform as-needed intravitreal injections of steroid
3. Increase the dose of prednisone to 1mg/kg/day
4. Refer to specialist for consideration of immunodulatory therapy

Your young patient with juvenile idiopathic arthritis and uveitis has been on varying doses of topical corticosteroid therapy for the last 3 months (now prednisolone acetate 1% BID OU). Today she has anterior chamber cells (2+) and the visual acuity is 20/70 due to cataract. You would:

1. Increase topical steroid to q1h and discuss eventual cataract surgery with the parents
2. Consider a periocular steroid injection under anesthesia
3. Discuss the case with the pediatric rheumatologist
4. a and c
